# *In Vitro* Comparison of Cytotoxicity and Genotoxicity of Three Vital Pulp Capping Materials

**DOI:** 10.22037/iej.v12i4.15104

**Published:** 2017

**Authors:** Azadeh Zakerzadeh, Ehsan Esnaashari, Sonia Dadfar

**Affiliations:** a *Department of Restorative, Dental School, Qazvin University of Medical Sciences & Health Services, Qazvin, Iran; *; b *Department of Endodontics, Dental Branch, Islamic Azad University, Tehran, Iran; *; c *Department of Operative Dentistry, Dental School, Gilan University of Medical Sciences, Rasht, Iran*

**Keywords:** Biodentine, Cytotoxicity, Genotoxicity, ProRoot MTA, TheraCal LC

## Abstract

**Introduction:**

Direct pulp capping (DPC) is a treatment for maintaining pulp vitality and its biological function. Ideally, pulp capping agents are expected to induce pulp cells to form hard tissue. This *in vitro* study assessed the cytotoxicity and genotoxicity of three vital pulp capping (VPC) agents naming Biodentine (Septodont, Saint-Maur-des-Fosses, France), mineral trioxide aggregate (ProRoot MTA; Dentsply, Tulsa Dental, Tulsa, OK, USA) and TheraCal LC (Bisco Inc, Schamburg, IL, USA) on human dental pulp fibroblasts.

**Methods and Materials:**

Human fibroblasts were exposed to 100 µL of ProRoot MTA, TheraCal LC and Biodentine in 0-1000 µg/mL concentrations and incubated at 37^°^C for 24 h. Their cytotoxicity and genotoxicity were assessed using the methyl thiazol tetrazolium (MTT) and the comet assays, respectively. The data were analyzed by Kruskal-Wallis test at the level of significance set at 0.05.

**Results:**

None of the tested materials had cytotoxicity or genotoxicity.

**Conclusion:**

TheraCal LC, Biodentine and ProRoot MTA can be alternately used for VPC treatment of teeth.

## Introduction

Restorative dental procedures aim to maintain pulp vitality in compromised teeth and decrease the need for root canal treatment. To achieve this goal, DPC and pulpotomy have been proposed [1, 2]. Direct pulp capping (DPC) is a method to cover the exposed pulp to maintain pulp vitality and its biological function. The ultimate goal of DPC is to induce mesenchymal pulp cells to differentiate form hard tissue [1]. Primarily, calcium hydroxide (CH)-based materials and today, mineral trioxide aggregate (MTA) have been generally recommended to seal the exposed pulp against oral cavity [3, 4]. It has been assumed that these materials cause primary changes to induce undifferentiated stem cells in the pulp tissue to differentiate into odontoblast-like cells with the ability to form a hard barrier at the site of exposure [5, 6]. Formation of tertiary reparative dentin in response to the application of CH is not only due to tissue induction by this agent but is also the result of the defense mechanisms of the pulp induced by the stimulatory function of CH [1, 7]. 

In endodontics, MTA has been successfully used for perforation repair and single-session apexification and pulp capping as well as a root end filling material [8, 9]. The clinical success of MTA is due to its optimal sealing ability [1], biocompatibility [1] and induction of odontoblast differentiation [7]. In contact with tissue fluids, it forms hydroxyapatite crystals [1]. TheraCal LC (Bisco Inc, Schamburg, IL, USA) is a light-cure modified resin. It is a calcium-silicate based liner for direct and indirect pulp capping. According to the manufacture, TheraCal LC is well tolerated by odontoblasts [1]. 

Biodentine (Septodont, Saint-Maur-des-Fosses, France) is a relatively new, biocompatible [10] calcium-silicate cement [9] and is a suitable alternative to CH-based materials [10]. It has been reported to have optimal biocompatibility and bio-induction. It is fast setting and has high compressive strength [9, 11]. 

Biocompatibility is a critical characteristic of pulp-capping materials. They must have antibacterial properties and optimal biocompatibility [12]. Moreover, they should induce healing and provide a hermetic seal. Biocompatibility is especially important since these materials are in direct contact with pulp tissue for long periods of time [13]. Biocompatible agents not only improve tissue healing but also organize the injured tissue [1, 14]. Assessment of cytotoxicity and genotoxicity is commonly performed to evaluate the biocompatibility of materials. Assessment of cytotoxicity is done to evaluate the biocompatibility of a material when exposed to cells [15]. Several methods have been proposed to determine the biocompatibility of dental materials [13, 16]. *In vitro* analysis of cell function is the first approach for this purpose [17]. Assessment of genotoxicity is particularly important for evaluation of biocompatibility as well [18]. Several systems have been developed to determine genotoxicity. Animal studies may be highly accurate for reflecting human metabolism; however, they are costly and time consuming. Thus, they are not often the first choice for assessment of genotoxicity and carcinogenesis of materials [15]. The MTT assay (3-{4,5-dimethylthiazol-2-yl}-2,5-diphenyl tetrazolium bromide3-(4.5-dimenthylthiazoyl)-2, 5-diphenyl-SH-tetrazelium bromide colorimetric assay, *aka* Mosmann’s Tetrazolium Toxicity assay) is a colorimetric assay for assessing cell viability. MTT, a yellow tetrazole, is absorbed by the mitochondria where it is reduced to purple formazan by succinate dehydrogenase in living cells. An acidified solution is added to dissolve the insoluble purple formazan product into a colored solution. The absorbance (optical density; OD) of this colored solution can be quantified by its measurement at a certain wavelength. By increased reduction of formazan and measurement of OD, cell viability and the cytotoxicity of materials can be measured.

Based on previous studies, the comet assay, also known as the single cell gel electrophoresis (SCGE), has been accepted as a simple and reliable technique for primary evaluation of genotoxicity [15, 19]. This method of assessment is based on migration of DNA in an agarose matrix and electrophoresis.

This study aimed to assess the cytotoxicity and genotoxicity of MTA, Biodentine and TheraCal LC against fibroblasts using the MTT and comet assays.

## Materials and Methods

Materials used in this study included ProRoot MTA (Dentsply, Tulsa Dental, Tulsa, OK, USA), Biodentine (Septodont, Saint Maurdes-Fosses, France) and TheraCal LC (Bisco Inc., Schaumburg, IL, USA), which were prepared in 7.8, 15.67, 31.25, 62.5, 125, 500 and 1000 μg/mL concentrations. 

***Cell culture***

Human dental pulp fibroblasts were cultured in Dulbecco’s modified Eagle’s medium (DMEM) (Dulbecco's Formula Modified, ICN Biochemicals, England) supplemented with 2 mM glutamine, 10% fetal bovine serum (FBS) and 100 units/mL penicillin or streptomycin. The culture medium was refreshed daily and incubated at 37 ^°^C with 5% CO_2_ and 95% oxygen. The cells were detached using a mixture of 0.25% trypsin or trypsin and EDTA. Some of the detached cells were cultured in the second culture medium [20]. 

***Assessment of cytotoxicity by the MTT assay***

The MTT assay was performed to measure the activity of enzymes that reduce MTT (SIGMA-Aldrich, St. Louis, MO, USA) and convert it to formazan dye, creating a purple color. Its main application is for the assessment of cell viability and proliferation. Also, it can be used to determine the cytotoxicity of pharmaceutical agents or toxic compounds since these materials prevent cell proliferation. 

The cells were placed in 96-well plates in such a way that each well contained 5000 cells and 100 μL of the respective material. After 24 h, the cells were exposed to ProRoot MTA (Dentsply, Tulsa Dental, Tulsa, OK, USA) Biodentine (Septodont, Saint-Maur-des-Fosses, France) and TheraCal LC (Bisco Inc, Schamburg, IL, USA). These materials were prepared according to the manufacturers’ instructions. Next, 0.05 mL of each mixture was separately added to wells containing extracellular solution.

Dilutions of the materials were prepared in 0-1000 μg/mL concentrations. Cultured untreated cells and cells treated with methyl methanesulfonate were considered as negative and positive controls, respectively. The MTT solution reached a final concentration of 0.05% using phosphate buffered saline and added to the mixture. After 2 h, formazan deposit was dissolved in dimethyl sulfoxide containing 10% glycine buffer with a pH of 5.10. Then, the microplates were gently shaken for 30 min in a dark room and absorbance was read at 570 and 620 nm wavelengths using a spectrophotometer (Biotek, Winoosky, VT, United States) [1, 19]. 

**Figure 1:**
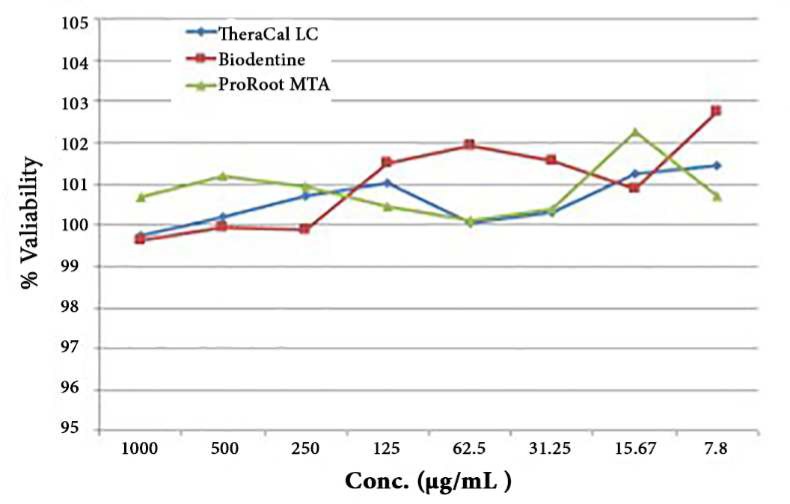
Cell viability after exposure to serial dilutions of test materials

***Assessment of genotoxicity by single cell-gel electrophoresis or the comet assay***

The alkaline SCGEA was done as described by Hosseinzadeh *et al.* [21, 22]. The cells were exposed to different concentrations of MTA, Biodentine and TheraCal and incubated for 24 h. The materials were prepared in 0-1000 μg/mL concentrations [23]. After elimination of materials, the cells were rinsed with cold phosphate buffered saline for three times and were then centrifuged at 3000 rpm at 4^°^ C for 5 min. Cotton pellets were dipped in phosphate buffered saline and placed on cells with 100000 seeding density. For the comet assay, 100 μL of normal melting point agar (Glen Burnie, MD, USA) was incrementally placed on the glass slide and covered with a cover slip. Next, the slides were placed on ice in order to convert agar into gel. Next, 10 μL of the suspension was mixed with 100 μL of low melting point agar (Glen Burnie, MD, USA) and after removal of cover slip, this mixture was incrementally applied on normal melting point agar. Another layer of low melting point agar was applied and the slide was immersed in cold lysing solution with a pH of 10 and stored at 0^°^ C in a dark room overnight. Next, the slides were placed in the electrophoresis apparatus (Paya Pajoohesh Pars, Tehran, Iran), covered with cold alkaline solution with a pH more than 13, incubated at 0^°^C in a dark room for 40 min and then electrophoresed at 0^°^C in a dark room for 30 min at 25 V and 300 mA. The slides were gently rinsed with 400 mM Trisma with its pH adjusted at 7.5 with hydrochloric acid for neutralization, stained with 50 μL of 20 μg/mL ethidium bromide (Merck Darmstadt, Germany) and covered again with a glass slab. 

For the comet assay, 100 nuclei of the two slides (each slide contained 50 nuclei) were selected and photographed under a fluorescence microscope (BX5O; Olympus, Tokyo, Japan) under ×400 magnification. This microscope had a 520-550 nm filter and a 580 nm barrier filter. Viable cells have intact nuclei and do not have a tail. Lysed cells have a comet-like appearance. DNA damage can be quantified by measuring the length of the comet tail and calculating the percentage of DNA in the tail using computerized image analysis software [2, 20]. 

***Data analysis***

The data were analyzed using SPSS software (version 20.0, SPSS, Chicago, IL, USA). The Kruskal Wallis test was first applied to compare cytotoxicity and genotoxicity of the three materials in different concentrations. Level of significance was set at 0.05.

## Results

Cytotoxicity of ProRoot MTA, Biodentine and TheraCal LC against fibroblasts was assessed using the MTT assay. Table 1 shows the dose-dependent response of each material in 0-1000 μg/mL concentrations (*P*=0.858). No statistically significant difference was noted among different concentrations of the three materials in terms of cytotoxicity (*P*=0.608) (Figure 1). Cells maintained their spindle shape after exposure to the materials. 

**Figure 2:**
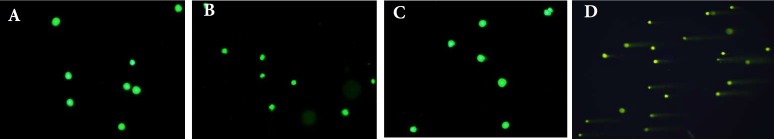
*A)* Image of non-damaged DNAs of Comet Assay in MTA group; *B)* Image of non-damaged DNAs of Comet Assay in Biodentine group; *C)* Image of non-damaged DNAs of Comet Assay in TheraCal LC group; *D)* Image of damaged DNAs of Comet Assay in positive Control

The comet assay was used to assess the genotoxicity (DNA damage in fibroblasts) of ProRoot MTA, Biodentine and TheraCal LC. DNA damage was quantified and reported as the percentage of DNA in the comet tail. The dose-dependent results of genotoxicity of the materials in 0-1000 μg/mL concentrations are shown in table 1 (*P*=0.105). No statistically significant difference was noted among the three materials in different concentrations in terms of genotoxicity (*P*=0.07) (Figure 2), and DNA of fibroblasts did not show a comet-like appearance after exposure to these materials.

## Discussion

*In vitro* studies are affordable and simple and provide valuable information. They can be performed under controlled conditions and can even explain the mechanism of cytotoxicity of materials [24]. The results of *in vitro* assessments may predict the probable effects *in vivo*. Cell culture studies are often used for assessment of cytotoxicity and genotoxicity of materials [2]. 

The MTT assay has been reported to be an efficient and accurate method for determination of cytotoxicity and quantification of cell viability under *in vitro* conditions [25, 26]. It is a colorimetric assay, which is based on the ability of the mitochondrial dehydrogenase enzyme of viable cells to convert water-soluble yellow tetrazolium salt to purple formazan crystals. The amount of generated formazan crystals has a direct association with the number of viable cells [2]. In other words, this test measures the number of viable cells based on the mitochondrial activity of the cells [27]. This method is easy, fast and accurate [2]. In the current study, cytotoxicity of the three materials in different concentrations was not significantly different. 

A previous study showed that viability of cells after exposure to diluted MTA and silicate cements was very high and no significant association was noted in terms of cytotoxicity among 10, 30 and 50 μg/mL concentrations after one day this result was in agreement with our findings [28]. Another study showed that Biodentine had the highest biocompatibility compared to MTA and TheraCal at 24, 48 and 72 h; MTA had optimal biocompatibility at the aforementioned time points as well. These results regarding the biocompatibility of Biodentine and MTA at 24 h were similar to our findings but cytotoxicity of TheraCal was significantly different from that of MTA and Biodentine at 24 h. TheraCal was cytotoxic only at 72 h, which was comparable with the cytotoxicity of CH [29]. Portland cement is the main component of TheraCal and its biocompatibility has been confirmed in several previous studies [20, 30]. This finding was in contrast to our results even at 24 h; such a controversy in the results may be attributed to the method of conduction of Alamar Blue test. Another study indicated that cytotoxicity of Biodentine and MTA was not significantly different in 1:1, 1:10 and 1:100 dilutions, which was in line with our results. However, TheraCal in aforementioned concentrations had significant differences with MTA and Biodentine in this regard. It was concluded that cytotoxicity of the three materials was dose-dependent and their cytotoxicity increased by an increase in their concentration. This finding was in contrast to our results, which may be attributed to the use of different tests (XTT assay in their study) and duration of study (three days) [31].

TheraCal LC pulp capping agent contains Portland cement, neutral resin monomers, hydrophobic resin monomers, hydrophilic fillers, photo-initiators and radiopacifiers. It is a light-cure single-component material. Released resin monomers, which have not been well polymerized, may accumulate and reach a toxic level over time. Thus, the cell irreversibly loses its glutathione metabolism as well as its other defense mechanisms and eventually undergoes apoptosis [32, 33]. The comet assay is a precise method for quantification of DNA

damage following exposure to genotoxic compounds [2]. Alkaline version of this test was used in the current study, which can determine the type of DNA damage and specify partially repaired areas [21, 22]. According to Kumaravel [34], tailed DNA percentage is an actual measure calculated by a computerized image analysis system, which determines the intensity and frequency of tails and tail length (length of DNA migration). This parameter is one of the most efficient measures to determine the induced DNA damage [35]. The comet assay is generally acceptable and has application for different types of cells [36]. Moroever, it has been shown that this test is sensitive and reliable for use *in vitro* [37]. 

The comet assay in the current study revealed that MTA, Biodentine and TheraCal LC did not cause DNA damage in any concentration. In a previous study, the precipitation assay was used for assessment of genotoxicity, which revealed that MTA and silicate cements in 10, 30 and 50 μg/mL concentrations did not cause DNA damage at one and seven days. This finding was in accordance with the current results [28]. Similarly, another study indicated that MTA and Portland cement in 1-1000 μg/mL concentrations had no cytotoxicity or genotoxicity against Chinese hamster ovary cells at 1 h after exposure at 37 ^°^C [38], which was in agreement with our results.

On the other hand, genotoxicity of MTA was evaluated in previous studies and the results revealed that MTA was not genotoxic for mouse lymphoma cells or human peripheral lymphocytes [22, 38]. However, some studies suggest that MTA is not neutral [39]. Balto *et al.* [40] assessed the morphology of human fibroblasts exposed to MTA and noticed that only a small number of cells remained viable and the remaining underwent structural changes. 

Another important factor in clinical application of pulp capping agents is the release of some components into the surrounding tissue, which is an advantage in the process of repair. It has been demonstarted that calcium oxide present in the composition of MTA-based cements can react with the tissue fluid and form CH [27]. Fridland and Rosado [41] identified calcium and OH ions as the most important components released from MTA. The latter ion is responsible for high alkalinity of MTA (pH=11). High pH induces fibroblast alkaline phosphatase, which is related to the process of mineralization [42]. Gandolfi *et al.* [43], showed that release of calcium from TheraCal LC was significantly higher than that from ProRoot MTA. Calcium ions play a fundamental role in different cellular bioactivities including the formation of mineralized tissue. High amounts of calcium ions can activate ATP and play an important role in the process of mineralization [44]. On the other hand, calcium ions induce the proliferation and differentiation of odontoblast-like cells [45]. It has been indicated that TheraCal LC can release OH ions for at least 28 days [46]. Furthermore, it has been proposed that the resin part of TheraCal LC can enhance the release of calcium and OH ions in humid environment [43]. The ability of TheraCal LC to release calcium ions and alkalinize the surrounding tissues is related to the formation of CH, which can be broken down into calcium and OH ions and raise the pH. The alkalinizing power of pulp capping agents is an essential criterion for their biological activities [43]. On the other hand, release of OH from TheraCal LC decreases after 7 to 14 days and the pH status returns to the physiological state. Thus, a humid environment is provided for the survival of pulp cells and their metabolic activity by the formation of new tertiary, reparative dentin [43]. 

**Table 1:** The mean (SD) of Genotoxic effects following exposure of fibroblast cells to MTA, Biodentine and TheraCal LC

**Concentration (µg** **/** **mL)**	**MTA**	**Biodentine**	**TheraCal LC**
7.8	0.50 (1.43)	0.71 (1.01)	1.10 (1.74)
15.67	0.45 (1.48)	0.80 (1.19)	1.15 (2.12)
31.25	0.56 (1.42)	0.81 (1.12)	1.10 (2.15)
62.5	1.72 (1.34)	1.12 (1.78)	0.44 (0.66)
125	0.56 (1.37)	0.61 (0.77)	1.78 (1.84)
250	0.15 (1.04)	0.54 (1.24)	1.06 (1.26)
500	0.28 (1.91)	0.95 (2.12)	1.45 (2.13)
1000	1.02 (2.10)	1.21 (1.79)	1.78 (2.59)

Biodentine is mainly composed of tri- and dicalcium silicate. Recent studies show that addition of tricalcium silicate to phosphate silicate enhances its bioactivity for odontoblasts and odontoblast-like cells, which may be related to the release of silicon (Si) from the calcium silicate cement [47]. Also, it has been well understood that Si has positive effects on bone metabolism. When released from biomaterials, it increases bone growth and osteogenesis [48]. Release of calcium and Si from Biodentine is greater than that from MTA [49]. Thus, MTA and Biodentine can form hydroxyapatite like surfaces in presence of body fluids containing calcium and phosphate. These surfaces are biocompatible and provide a suitable environment for cell adhesion and proliferation [50]. Moreover, Biodentine can induce the differentiation of progenitor cells into odontoblasts and create a mineralized matrix possessing dentin properties [51]. 

Thus, it can be concluded that Biodentine and TheraCal LC are not significantly different from MTA in terms of cytotoxicity or genotoxicity. Since Biodentine has a setting time of 9 to 12 min and TheraCal LC has simple application since it is supplied in the form of a syringe with a small tip, they can be suitable alternatives to MTA for DPC since MTA has a long setting time and difficult handling. 

However, it should be noted that results of primary genotoxicity tests have some limitations and future studies on TheraCal LC and Biodentine are required to further assess their genotoxicity by use of methods such as quantitative real time polymerase chain reaction, micronucleus and necrosis tests and apoptosis assay by FACS.

## Conclusion

ProRoot MTA, Biodentine and TheraCal LC did not show cytotoxicity or genotoxicity in 0-1000 μg/mL concentrations. Considering the short setting time of Biodentine and simple use of TheraCal LC, they are suitable alternatives to ProRoot MTA for DPC.

## References

[1] Poggio C, Arciola CR, Beltrami R, Monaco A, Dagna A, Lombardini M, Visai L (2014). Cytocompatibility and antibacterial properties of capping materials. ScientificWorldJournal.

[2] Naghavi N, Ghoddusi J, Sadeghnia HR, Asadpour E, Asgary S (2014). Genotoxicity and cytotoxicity of mineral trioxide aggregate and calcium enriched mixture cements on L929 mouse fibroblast cells. Dent Mater J.

[3] Prati C, Siboni F, Polimeni A, Bossu M, Gandolfi MG (2014). Use of calcium-containing endodontic sealers as apical barrier in fluid-contaminated wide-open apices. J Appl Biomater.

[4] Malik G, Bogra P, Singh S, Samra RK (2013). Comparative evaluation of intracanal sealing ability of mineral trioxide aggregate and glass ionomer cement: An in vitro study. J Conserv Dent.

[5] Saberi EA, Karkehabadi H, Mollashahi NF (2016). Cytotoxicity of Various Endodontic Materials on Stem Cells of Human Apical Papilla. Iran Endod J.

[6] Laurent P, Camps J, About I (2012). BiodentineTM induces TGF‐β1 release from human pulp cells and early dental pulp mineralization. Int Endod J.

[7] Nosrat A, Peimani A, Asgary S (2013). A preliminary report on histological outcome of pulpotomy with endodontic biomaterials vs calcium hydroxide. Restor Dent Endod.

[8] Chang S-W, Lee S-Y, Ann H-J, Kum K-Y, Kim E-C (2014). Effects of calcium silicate endodontic cements on biocompatibility and mineralization-inducing potentials in human dental pulp cells. J Endod.

[9] Khedmat S, Dehghan S, Hadjati J, Masoumi F, Nekoofar MH, Dummer PMH (2014). In vitro cytotoxicity of four calcium silicate-based endodontic cements on human monocytes, a colorimetric MTT assay. Restor Dent Endod.

[10] Zanini M, Sautier JM, Berdal A, Simon S (2012). Biodentine induces immortalized murine pulp cell differentiation into odontoblast-like cells and stimulates biomineralization. J Endod.

[11] Grech L, Mallia B, Camilleri J (2013). Investigation of the physical properties of tricalcium silicate cement-based root-end filling materials. Dent Mater.

[12] Jaberiansari Z, Naderi S, Tabatabaei FS (2014). Cytotoxic effects of various mineral trioxide aggregate formulations, calcium-enriched mixture and a new cement on human pulp stem cells. Iran Endod J.

[13] Saygili G, Saygili S, Tuglu I, Davut Capar I (2017). In Vitro Cytotoxicity of GuttaFlow Bioseal, GuttaFlow 2, AH-Plus and MTA Fillapex. Iran Endod J.

[14] Braz MG, Camargo E, Salvadori DMF, Marques M, Ribeiro D (2006). Evaluation of genetic damage in human peripheral lymphocytes exposed to mineral trioxide aggregate and Portland cements. J Oral Rehabil.

[15] Maria de Lourdes RA, Holland R, Reis A, Bortoluzzi MC, Murata SS, Dezan E, Souza V, Alessandro LD (2008). Evaluation of mineral trioxide aggregate and calcium hydroxide cement as pulp-capping agents in human teeth. J Endod.

[16] Farhad Mollashahi N, Saberi E, Karkehabadi H (2016). Evaluation of Cytotoxic Effects of Various Endodontic Irrigation Solutions on the Survival of Stem Cell of Human Apical Papilla. Iran Endod J.

[17] Mantellini M, Botero T, Yaman P, Dennison J, Hanks C, Nör J (2003). Adhesive resin induces apoptosis and cell-cycle arrest of pulp cells. J Dent Res.

[18] Zeferino E, Bueno C, Oyama L, Ribeiro D (2010). Ex vivo assessment of genotoxicity and cytotoxicity in murine fibroblasts exposed to white MTA or white Portland cement with 15% bismuth oxide. Int Endod J.

[19] Schmalz G, Schweikl H (1994). Characterization of an in vitro dentin barrier test using a standard toxicant. J Endod.

[20] Zeferino E, Bueno C, Oyama L, Ribeiro D (2010). Ex vivo assessment of genotoxicity and cytotoxicity in murine fibroblasts exposed to white MTA or white Portland cement with 15% bismuth oxide. International endodontic journal.

[21] Hosseinzadeh H, Abootorabi A, Sadeghnia HR (2008). Protective effect of crocus sativus stigma extract and crocin (trans-crocin 4) on Methyl Methanesulfonate–induced DNA damage in mice organs. DNA and cell biology.

[22] Hosseinzadeh H, Sadeghnia HR (2007). Effect of safranal, a constituent of Crocus sativus (Saffron), on methyl methanesulfonate (MMS)–induced DNA damage in mouse organs: an alkaline single-cell gel electrophoresis (Comet) assay. DNA and cell biology.

[23] Matsuo T, Nakanishi T, Shimizu H, Ebisu S (1996). A clinical study of direct pulp capping applied to carious-exposed pulps. J Endod.

[24] Ribeiro DA, Matsumoto MA, Duarte MAH, Marques MEA, Salvadori DMF (2005). In vitro biocompatibility tests of two commercial types of mineral trioxide aggregate. Braz Oral Res.

[25] Camilleri J (2008). The biocompatibility of modified experimental Portland cements with potential for use in dentistry. Int Endod J.

[26] Chiang T-Y, Ding S-J (2010). Comparative physicochemical and biocompatible properties of radiopaque dicalcium silicate cement and mineral trioxide aggregate. J Endod.

[27] Bin CV, Valera MC, Camargo SE, Rabelo SB, Silva GO, Balducci I, Camargo CHR (2012). Cytotoxicity and genotoxicity of root canal sealers based on mineral trioxide aggregate. J Endod.

[28] Ding S-J, Kao C-T, Chen C-L, Shie M-Y, Huang T-H (2010). Evaluation of human osteosarcoma cell line genotoxicity effects of mineral trixoide aggregate and calcium silicate cements. J Endod.

[29] Poggio C, Ceci M, Dagna A, Beltrami R, Colombo M, Chiesa M (2015). In vitro cytotoxicity evaluation of different pulp capping materials: a comparative study. Arh Hig Rada Toksikol.

[30] Min K-S, Kim H-I, Park H-J, Pi S-H, Hong C-U, Kim E-C (2007). Human pulp cells response to Portland cement in vitro. J Endod.

[31] Bortoluzzi EA, Niu L-n, Palani CD, El-Awady AR, Hammond BD, Pei D-d, Tian F-c, Cutler CW, Pashley DH, Tay FR (2015). Cytotoxicity and osteogenic potential of silicate calcium cements as potential protective materials for pulpal revascularization. Dent Mater.

[32] Engelmann J, Janke V, Volk J, Leyhausen G, Von Neuhoff N, Schlegelberger B, Geurtsen W (2004). Effects of BisGMA on glutathione metabolism and apoptosis in human gingival fibroblasts in vitro. Biomaterials.

[33] Engelmann J, Leyhausen G, Leibfritz D, Geurtsen W (2001). Metabolic effects of dental resin components in vitro detected by NMR spectroscopy. J Dent Res.

[34] Kumaravel T, Jha AN (2006). Reliable Comet assay measurements for detecting DNA damage induced by ionising radiation and chemicals. Mutat Res.

[35] Tice R, Agurell E, Anderson D, Burlinson B, Hartmann A, Kobayashi H, Miyamae Y, Rojas E, Ryu J, Sasaki Y (2000). Single cell gel/comet assay: guidelines for in vitro and in vivo genetic toxicology testing. Environ Mol Mutagen.

[36] Kleinsasser NH, Schmid K, Sassen AW, Harréus UA, Staudenmaier R, Folwaczny M, Glas J, Reichl F-X (2006). Cytotoxic and genotoxic effects of resin monomers in human salivary gland tissue and lymphocytes as assessed by the single cell microgel electrophoresis (Comet) assay. Biomaterials.

[37] McKelvey-Martin V, Green M, Schmezer P, Pool-Zobel B, De Meo M, Collins A (1993). The single cell gel electrophoresis assay (comet assay): a European review. Mutat Res.

[38] Ribeiro DA, Sugui MM, Matsumoto MA, Duarte MAH, Marques MEA, Salvadori DMF (2006). Genotoxicity and cytotoxicity of mineral trioxide aggregate and regular and white Portland cements on Chinese hamster ovary (CHO) cells in vitro. Oral Surg Oral Med Oral Pathol Oral Radiol Endod.

[39] Hirschman WR, Wheater MA, Bringas JS, Hoen MM (2012). Cytotoxicity comparison of three current direct pulp-capping agents with a new bioceramic root repair putty. J Endod.

[40] Balto HA (2004). Attachment and morphological behavior of human periodontal ligament fibroblasts to mineral trioxide aggregate: a scanning electron microscope study. J Endod.

[41] Fridland M, Rosado R (2003). Mineral trioxide aggregate (MTA) solubility and porosity with different water-to-powder ratios. J Endod.

[42] Lee B-N, Son H-J, Noh H-J, Koh J-T, Chang H-S, Hwang I-N, Hwang Y-C, Oh W-M (2012). Cytotoxicity of newly developed ortho MTA root-end filling materials. J Endod.

[43] Gandolfi M, Siboni F, Prati C (2012). Chemical–physical properties of TheraCal, a novel light‐curable MTA‐like material for pulp capping. Int Endod J.

[44] Torneck C, Moe H, Howley T (1983). The effect of calcium hydroxide on porcine pulp fibroblasts in vitro. J Endod.

[45] Danesh G, Dammaschke T, Gerth H, Zandbiglari T, Schäfer E (2006). A comparative study of selected properties of ProRoot mineral trioxide aggregate and two Portland cements. Int Endod J.

[46] Shubich I, Miklos FL, Rapp R, Draus FJ (1978). Release of calcium ions from pulp-capping materials. J Endod.

[47] Jung S, Mielert J, Kleinheinz J, Dammaschke T (2014). Human oral cells’ response to different endodontic restorative materials: an in vitro study. Head & face medicine.

[48] Morejón‐Alonso L, Ferreira OJB, Carrodeguas RG, dos Santos LA (2012). Bioactive composite bone cement based on α‐tricalcium phosphate/tricalcium silicate. J Biomed Mater Res B Appl Biomater.

[49] Han L, Okiji T (2013). Bioactivity evaluation of three calcium silicate‐based endodontic materials. Int Endod J.

[50] Patel N, Best S, Bonfield W, Gibson I, Hing K, Damien E, Revell P (2002). A comparative study on the in vivo behavior of hydroxyapatite and silicon substituted hydroxyapatite granules. J Mater Sci Mater Med.

[51] Schmalz G, Galler K, About I (2011). Dentin regeneration in vitro: the pivotal role of supportive cells. Adv Dent Res.

